# Concomitant associations of dietary behaviours and physical activity with body composition in adolescents

**DOI:** 10.1186/s12889-025-25642-4

**Published:** 2025-12-24

**Authors:** Beáta Ružbarská, Peter Bakalár, Monika Vašková, Jaroslava Kopčáková, Peter Kolarčik, José Francisco López-Gil

**Affiliations:** 1 University of Prešov, Faculty of Sports, Department of Sports Educology and Humanistics, 17. novembra 15, Prešov, 08001 Slovakia; 2 Palacký University Olomouc, Faculty of Physical Culture, Institute of Active Lifestyle, tř. Míru 117, Olomouc, 771 47 Czechia; 3 Pavol Jozef Šafárik University, Faculty of Medicine, Medical Education Centre, Trieda SNP 1, Košice, 04011 Slovakia; 4 Palacký University Olomouc, Olomouc University Social Health Institute, Univerzitni 22, Olomouc, 771 11 Czechia; 5 Pavol Jozef Šafárik University, Faculty of Medicine, Department of Health Psychology and Research Methodology, Trieda SNP 1, Košice, 04011 Slovakia; 6 Universidad Espíritu Santo, School of Medicine, Av. Samborondón 5, Samborondón, 092301 Ecuador; 7 Universidad de Los Lagos, Vicerrectoría de Investigación y Postgrado, Lord Cochrane 1070, Osorno, Región de Los Lagos Chile

**Keywords:** Eating behaviour, Movement behaviour, Body mass index, Percentage of body fat, Visceral fat area

## Abstract

**Background:**

Development of adolescent obesity is associated with substantial health risks and comorbidities and thus represents a major public health concern. This study aimed to examine the associations between dietary behaviours and physical activity with body composition indicators among Slovak adolescents.

**Methods:**

We used data from the Health Behaviour in School-Aged Children (HBSC) study conducted in 2022 in Slovakia. The sample consisted of 1225 adolescents (mean age 13.3 ± 1.4; 53.9% boys). The HBSC questionnaire items were used to assess frequency of fruits, vegetables, sweets, soft and energy drinks, breakfast consumption during weekdays, having breakfast with family, moderate-to-vigorous physical activity (MVPA) and vigorous physical activity (VPA). Body height (cm) was measured using a SECA 213 portable stadiometer, and body composition was assessed via bioimpedance analysis (InBody 230). Linear regression was used to test associations between dietary behaviours and physical activity variables and body composition outcomes.

**Results:**

More frequent breakfast eating during weekdays and more frequent sweets consumption were inversely associated with body mass index (BMI), percentage of body fat (PBF), visceral fat area (VFA) and fat-free mass index (FFMI). Higher MVPA was inversely associated with BMI, PBF and VFA but positively associated with FFMI. Greater VPA was associated with PBF and VFA, but inversely associated with FFMI.

**Conclusions:**

These findings suggest that more frequent breakfast consumption and moderate-to-vigorous physical activity are associated with favourable body composition parameters in adolescents.

## Background

Endocrine-regulated changes in body composition are a normal feature of adolescence [1]. Nevertheless, under the influence of various external factors, adverse changes may occur, leading to the development of adolescent obesity, which is associated with substantial health risks and comorbidities and thus represents a major public health concern [2]. According to the latest data, the global age-standardised prevalence of obesity in school-aged children and adolescents increased markedly from 1990 to 2022 in both girls (1.7% vs. 6.9%) and boys (2.1% vs. 9.3%) [3]. This trend is alarming, as evidence indicates that obesity during adolescence frequently persists into adulthood and is consequently associated with excess adult mortality [4]. Furthermore, upward transitions across BMI categories from adolescence to adulthood are more common than reversals, suggesting that preventive pathways may be easier to identify than those leading to weight reduction [5]. The most prevalent cause of obesity in childhood and adolescence is an imbalance between energy intake and expenditure - namely, excessive caloric intake unaccompanied by adequate energy expenditure [6]. The primary determinants of this imbalance include behavioural factors such as dietary habits (diet quality, frequent fast-food consumption, eating occasions away from home, large portion sizes, high intake of sugar-sweetened beverages, and breakfast omission) and movement behaviours (sedentary behaviour and physical activity) [2, 7]. Recent epidemiological data from the Health Behaviour in School-aged Children (HBSC) study [8] show that only 51% of adolescents consume breakfast daily on weekdays; 38% eat fruit daily; 38% report daily vegetable consumption; 25% consume sweets or chocolate daily; and 15% report daily consumption of sugary soft drinks. Concurrently, only 25% of boys and 15% of girls achieve 60 min of moderate-to-vigorous physical activity per day, although 60% of adolescents meet the WHO recommendation for engaging in vigorous physical activity at least three times per week. These findings are notable in the context of previous evidence that vigorous physical activity is more strongly associated with reduced total and central adiposity compared with moderate or combined moderate-to-vigorous activity, while sedentary behaviour remains a significant predictor of unfavourable body composition parameters [9, 10].

Given that dietary and movement behaviour factors may interact in either antagonistic or synergistic ways in influencing body composition, it is essential to examine their associations with body composition concurrently [11]. For example, physically active adolescents tend to exhibit more favourable body composition, higher physical fitness, and healthier dietary behaviours compared with their less active peers, who may in turn develop restrictive dietary patterns that contribute to overeating and weight gain [12, 13]. Conversely, higher levels of physical activity may foster the belief that energy intake need not be controlled, as the adverse consequences of unhealthy eating can ostensibly be compensated by exercise [14].

Recent Central European studies further corroborate this interrelation, indicating that dietary patterns and physical activity tend to cluster and jointly influence adolescent body composition: adolescents who are physically active and adhere to healthier diets typically exhibit lower adiposity, whereas low activity combined with unhealthy eating is associated with higher BMI and central fat accumulation [15–17].

Therefore, the aim of this study was to examine the concomitant associations of dietary behaviours and physical activity with body composition based on the data from Slovakian adolescents.

## Methods

### Sample and procedure

Data were obtained from the 2022 Slovak wave of the Health Behaviour in School-aged Children (HBSC) survey using a three-stage sampling design. First, 195 elementary schools were randomly selected from the national registry of eligible schools provided by the Slovak Institute of Information and Prognosis for Education; 94 schools agreed to participate (school response rate 48%). Second, survey data were collected from 9,697 students in grades 5–9 (mean age 13.4 ± 1.3 years; 50.9% boys). Third, a regional subsample was drawn from 11 elementary schools and one eight-year grammar school in the Prešov self-governing region (≈ 13% of participating schools). After excluding incomplete questionnaires, the final analytic sample comprised 1,225 adolescents (mean age 13.3 ± 1.4 years; 53.9% boys).

The study protocol was approved by the Ethics Committee of the Medical Faculty, P. J. Safarik University in Košice (approval no. 13/N2021). Parental consent was obtained using a passive (opt-out) procedure, whereby parents were informed about the study by the school administration and could decline their child’s participation. Students were informed in advance by their teachers and again at the time of data collection by HBSC administrators, who emphasized their right to refuse participation. Participation was entirely voluntary, anonymous, and conducted without incentives, and students’ assent was obtained by their decision to participate.

### Measures

#### Dietary behaviours

Dietary behaviours were assessed using items from the HBSC questionnaire [18], focusing on the frequency of fruit, vegetable, sweet, and sugar-sweetened beverage consumption, as well as breakfast habits and family meals.

HBSC dietary items demonstrate measurement properties that are appropriate for population surveillance. The dietary food-frequency items show moderate to substantial test–retest reliability, with weighted kappa values ranging from 0.43 to 0.70 and Spearman correlations between 0.52 and 0.82. Relative validity testing against 7-day food diaries and 24-hour checklists reports correlations ranging from 0.10 to 0.65, indicating that the items perform well for ranking adolescents according to frequency of dietary behaviours, although they are less suitable for estimating precise intake because adolescents tend to over-report consumption in the questionnaire [19, 20].

For other HBSC dietary behaviour items – breakfast consumption and family meals – test–retest analyses indicate acceptable reliability (classified as sufficient in validation studies). However, for these items, validity evidence is limited, as few studies have compared them directly to external reference methods, and specific validity coefficients are not consistently reported [20].

Food intake was measured with the item: *“How many times a week do you usually eat or drink… (fruits; vegetables; sweets such as candy or chocolate; Coke or other sugar-sweetened soft drinks; energy drinks*, e.g.,* Red Bull)?”* Response options included: never; less than once a week; once a week; 2–4 days per week; 5–6 days per week; once daily; more than once daily.

Breakfast consumption during weekdays was assessed with the item: *“How often do you usually have breakfast (more than just a glass of milk or fruit juice)?”* Response categories were: never; 1 day; 2 days; 3 days; 4 days; 5 days.

Family meals were assessed by the question: *“How often do you and your family usually have meals together?”* with response options: every day; most days; about once a week; less often; never.

### Movement behaviours

#### Moderate-to-vigorous physical activity (MVPA)

MVPA was assessed using an item adapted for the HBSC study from the original instrument developed by Prochaska et al. [21] for use in adolescent clinical practice. Its validity was established against seven-day continuous accelerometer measurement (*r* = 0.40, *p* < 0.001), and it demonstrated substantial test–retest reliability (ICC = 0.77). More recent studies show intraclass correlation coefficients (ICC) ranging from 0.41 to 0.60, and up to ICC = 0.74 in some samples, indicating consistent reporting over repeated measurements [22, 23]. Criterion validity against accelerometry demonstrates moderate correlations of *r* = 0.32–0.44, supporting its ability to capture relative differences in adolescents’ activity levels [23, 24].

The item asked: *“Over the past 7 days*,* on how many days were you physically active for a total of at least 60 minutes per day? Please add up all the time you spent in physical activity each day.”* Response options ranged from 0 to 7 days.

#### Vigorous physical activity (VPA)

VPA was defined as physical activity performed during leisure time outside school that leads to sweating or breathlessness [8]. This HBSC item shows moderate test–retest reliability within a similar ICC range (0.41–0.60), and construct validity has been demonstrated through significantly higher fitness levels among adolescents classified as active [22, 25].

The item asked: *“Outside school hours: How often do you usually exercise in your free time so much that you get out of breath or sweat?”* Response categories were: every day, 4–6 times per week, 3 times per week, 2 times per week, once per week, less than once per month, and never.

Overall, while MVPA shows stronger correlations with accelerometer-measured activity than VPA, both measures are appropriate for ranking adolescents by activity level and monitoring population trends.

### Body composition

Body height (cm) was measured with a SECA 213 portable stadiometer. Participants stood barefoot in an upright position with their backs against the device and heads oriented in the Frankfurt horizontal plane. Measurements were taken during inspiration and recorded to the nearest 0.1 mm.

Body weight (kg) was measured simultaneously with body mass index (BMI), percentage of body fat (PBF), and visceral fat area (VFA) during bioelectrical impedance analysis (BIA) using InBody 230 device (InBody Co., Seoul, Korea). This device demonstrated strong predictive capacity with excellent discriminatory ability (AUC = 0.964 with protocol, 0.973 without protocol; sensitivity = 96.8% and 88.4%; specificity = 86.7% and 95.8%, respectively) and substantial agreement with DXA (kappa index = 0.67 with protocol and 0.63 without protocol, both *p* < 0.001) for assessing body fat in adolescents, performing well both with and without a standardized protocol, making it a suitable option for population studies where strict protocol adherence might be difficult [26]. To account for clothing, students were instructed to wear light sportswear (e.g., T-shirt and pants/skirt), and a correction factor of − 0.3 kg was applied. Fat-free mass index (FFMI, kg/m²) was subsequently calculated using the measured data [27].

### Data analysis

As a first step, we described the main variables using frequency tables. We calculated means and standard deviations for continual variables and prevalence for categorical variables. The normality distribution of the assessed variables was tested using the Kolmogorov-Smirnov and Shapiro-Wilk test while refusing the normality hypothesis. This was the reason for using non-parametric test in cross-sectional comparisons. We also tested differences between boys and girls using Mann-Whitney U-test for continual variables and Pearson chi^2^ test for categorical variables. Simple linear regression procedures (with Gaussian distribution of dependents variables) were used to test associations between dietary behaviours and physical activity variables with body composition outcomes (BMI, PBF, VFA and FFMI). Variance Inflation Factor (VIF) was used for multicollinearity assessment (threshold above 5 was used as an indicator of multicollinearity), residual normality was evaluated through visual inspection of Q-Q plots and the Shapiro-Wilk test. The results however did not support the assumption of normality of residuals in each dependent variable. Unstandardized regression beta coefficients (Β) and their 95% confidence intervals (CIs) were determined from the full adjusted models.

Statistical analyses were processed using statistical software packages IBM SPSS 25.0 (IBM Corp. Released 2017. IBM SPSS Statistics for Windows, Version 25.0. Armonk, NY: IBM Corp.).

## Results

Descriptive statistics of the sample showed statistically significant differences between boys and girls in most body composition variables except VFA (Table 1). Boys had higher values in BMI, FFMI and BMI, in the other hand the girls had higher values of PBF. Physical activity was significantly more frequent among boys compared to girls (MVPA and VPA). Regarding dietary behaviours, we found statistically significant differences in reported frequency in all items.

As mentioned above, boys had higher values in BMI (median(IQR): 19.6(17.5–22.6) vs. 19.3(17.3–21.6); *p* < 0.05) and FFMI (median(IQR): 16.2(14.7–17.9 vs. 14.8(13.9–15.8); *p* < 0.001), on the other hand the girls had higher values of PBF (median(IQR): 23.0(18.0–28.4.0.4) vs. 15.5(10.5–24.8); *p* < 0.001). Physical activity was significantly more frequent among boys compared to girls, both MVPA (median(IQR): 5.0(3.0–6.0) vs. 4.0(3.0–6.0); *p* < 0.001) and VPA (median(IQR): 2.0(1.0–3.0) vs. 3.0(2.0–5.0; *p* < 0.001). Regarding dietary behaviours, we found statistically significant differences in reported frequency in all items. Boys reported more frequently eating breakfast in weekdays (median(IQR): 2.0(1.0–3.0) vs. 3.0(2.0–5.0); *p* < 0.05) and drinking soft drinks (median/IQR: 4.0(2.0–5.0) vs. 3.0(2.0–4.0); *p* < 0.001) and energy drinks (median(IQR): 1.0(1.0–2.0) vs. 1.0(1.0–2.0); *p* < 0.001). Girls reported more frequently eating meals with family (median(IQR): 2.0(2.0–3.0) vs. 2.0 (1.0–3.0); *p* < 0.001), eating fruits (median(IQR): 5.0(4.0–6.0) vs. 4.0(4.0–6.0); *p* < 0.01), vegetables (median(IQR): 5.0(4.0–6.0) vs. 4.0(3.0–6.0); *p* < 0.001) and sweets (median(IQR): 5.0(4.0–6.0) vs. 4.0(3.0–6.0); *p* < 0.001) compared to boys.

**Table 1:** Body composition indicators, dietary behaviours and physical activity variables in whole sample and compared between boys and girls (Mann Whitney U-test)

	Total sample		Boys		Girls		Diference statistics U-test value
	Median (IQR)	M (SD)	Median (IQR)	M (SD)	Median (IQR)	M (SD)	
**BMI**	19.5 (17.4–22.2)	*20.2 (3.9)*	19.6 (17.5–22.6)	*20.4 (4.0)*	19.3 (17.3–21.6)	*19.9 (3.7)*	169685.50 *
**PBF**	20.2 (13.5–27.4)	*21.0 (9.6)*	15.5 (10.5–24.8)	*18.3 (9.7)*	23.0 (18.0–28.4.0.4)	*23.9 (8.1)*	113769.50 ***
**VFA**	35.9 (20.5–59.9)	*44.5 (32.5)*	34.0 (17.7–61.3)	*43.7 (33.3)*	36.5 (23.9–57.6)	*44.4 (29.6)*	*171579.00*
**FFMI**	15.5 (14.2–16.8)	*15.7 (2.0)*	16.2 (14.7–17.9)	*16.4 (2.2)*	14.8 (13.9–15.8)	*14.9 (1.5)*	107009.50 ***
**Breakfast weekdays**	4.0 (2.0–6.0)	*3.9 (2.1)*	5.0 (2.0–6.0)	*4.1 (2.0)*	4.0 (1.0–6.0)	*3.8 (2.1)*	161309.50 *
**Family meal**	2.0 (1.0–3.0)	*2.3 (1.2)*	2.0 (1.0–3.0)	*2.1 (1.1)*	2.0 (2.0–3.0)	*2.5 (1.2)*	144632.00 ***
**Fruits**	5.0 (4.0–6.0)	*4.8 (1.6)*	4.0 (4.0–6.0)	*4.6 (1.6)*	5.0 (4.0–6.0)	*4.9 (1.5)*	157707.50 **
**Vegetables**	4.0 (3.0–6.0)	*4.5 (1.6)*	4.0 (3.0–6.0)	*4.3 (1.6)*	5.0 (4.0–6.0)	*4.6 (1.6)*	148622.50 ***
**Sweets**	4.0 (3.0–6.0)	*4.6 (1.6)*	4.0 (3.0–6.0)	*4.4 (1.7)*	5.0 (4.0–6.0)	*4.8 (1.6)*	147817.50 ***
**Soft drinks**	3.0 (2.0–5.0)	*3.6 (1.8)*	4.0 (2.0–5.0)	*3.7 (1.8)*	3.0 (2.0–4.0)	*3.4 (1.7)*	148874.50 ***
**Energy drinks**	1.0 (1.0–2.0)	*1.7 (1.4)*	1.0 (1.0–2.0)	*1.8 (1.5)*	1.0 (1.0–2.0)	*1.6 (1.3)*	154368.50 ***
**MVPA**	4.0 (3.0–6.0)	*4.3 (2.0)*	5.0 (3.0–6.0)	*4.5 (2.1)*	4.0 (3.0–6.0)	*4.1 (2.0)*	157874.00 ***
**VPA**	2.0 (2.0–4.0)	*3.0 (1.9)*	2.0 (1.0–3.0)	*2.7 (1.8)*	3.0 (2.0–5.0)	*3.4 (2.0)*	123879.50 ***

*BMI* Body mass index, *PBF* Percentage of body fat, *VFA* Visceral fat area, *FFMI* Fat-free mass index (kg/m²), *MVPA* Moderate-to-vigorous physical activity, *VPA* Vigorous physical activity, *M* mean, *SD* Standard deviation, *IQR* Interquartile range, *p < 0.05, **p < 0.01, ***, *p* < 0.001

Regression analysis showed, that from the individually tested dietary behaviours with each of the body compositions variables, only few of them proved to be significantly associated with them (Table 2). We found that more frequent breakfast eating during weekdays is negatively associated with BMI (B(95%CI)=−0.19(−0.29 | −0.08); *p* < 0.001), PBF (B(95%CI)=−0.37(−0.63 | −0.11); *p* < 0.01), VFA (B(95%CI)=−1.27(−2.16 | −0.39); *p* < 0.01) and FFMI (B(95%CI)=−0.07 (−0.13 | −0.02); *p* < 0.05). Similarly, only one more dietary behaviour, sweets consumption, was associated with all four body composition variables. We found that more frequent sweets consumption is negatively associated with BMI, PBF, VFA and FFMI (B(95%I)=−0.38(−0.51 | −0.24); −0.74(−1.07 | −0.41); −2.67 (−3.78 | −1.56); −0.14 (−0.21 | −0.07) respectively; all *p* < 0.001). Furthermore, we found more frequent energy drinks consumption negatively associated with PBF (B(95%CI)=−0.66(−1.05 | −0.27); *p* < 0.001) but positively with FFMI (B(95%CI) = 0.25(0.17 | 0.34); *p* < 0.001). Soft drinks consumption is negatively associated only with PBF variable (B(95%CI)=−0.44(−0.75 | −0.12); *p* < 0.01). Remaining dietary behaviours, specifically the reported frequency of having breakfast with family, frequency of fruit and vegetable consumption showed no statistically significant association with any of four tested body composition variables (BMI, PBF, VFA, FFMI).

**Table 2:** Associations of dietary behaviours, physical activity variables on body composition variables as crude effect (linear regression)

Crude effects	BMI	PBF	VFA	FFMI
	B (95% C.I.)	B (95% C.I.)	B (95% C.I.)	B (95% C.I.)
**Breakfast weekdays**	−0.19 (−0.29 | −0.08) ***	−0.37 (−0.63 | −0.11) **	−1.27 (−2.16 | −0.39) **	−0.07 (−0.13 | −0.02) *
**Family meal**	0.13 (−0.06 | 0.32)	0.23 (−0.23 | 0.68)	−0.11 (−1.65 | 1.43)	0.07 (−0.03 | 0.17)
**Fruits**	−0.09 (−0.23 | 0.05)	0.01 (−0.34 | 0.35)	−0.98 (−2.14 | 0.18)	−0.07 (−0.15 | 0.00)
**Vegetables**	0.01 (−0.12 | 0.15)	0.19 (−0.14 | 0.53)	0.11 (−1.02 | 1.24)	−0.03 (−0.1 | 0.05)
**Sweets**	−0.38 (−0.51 | −0.24) ***	−0.74 (−1.07 | −0.41) ***	−2.67 (−3.78 | −1.56) ***	−0.14 (−0.21 | −0.07) ***
**Soft drinks**	−0.05 (−0.18 | 0.07)	−0.44 (−0.75 | −0.12) **	−0.51 (−1.55 | 0.54)	0.04 (−0.02 | 0.11)
**Energy drinks**	0.13 (−0.03 | 0.29)	−0.66 (−1.05 | −0.27) ***	−0.38 (−1.69 | 0.92)	0.25 (0.17 | 0.34) ***
**MVPA**	−0.12 (−0.22 | −0.01) *	−0.81 (−1.07 | −0.55) ***	−1.96 (−2.83 | −1.08) ***	0.07 (0.02 | 0.13) **
**VPA**	0.09 (−0.02 | 0.21)	0.72 (0.44 | 1.00) ***	1.77 (0.81 | 2.72) ***	−0.07 (−0.14 | −0.01) *
**Sex** (boys vs. girls)	−0.53 (−0.96 | −0.10)	5.57 (4.55 | 6.60) ***	0.76 (−2.82 | 4.33)	−1.53 (−1.74 | −1.31) ***
**Age**	0.56 (0.41 | 0.70) ***	−0.82 (−1.19 | −0.46) ***	0.36 (−0.87 | 1.59)	0.64 (0.57 | 0.71) ***

*BMI* Body mass index, *PBF* Percentage of body fat, *VFA* Visceral fat area, *FFMI* Fat-free mass index (kg/m²), *MVPA* Moderate-to-vigorous physical activity, *VPA* Vigorous physical activity, *p < 0.05, **p < 0.01, ***, *p* < 0.001

Regarding physical activity variables, MVPA and VPA, we found MVPA being negatively associated with BMI, PBF and VFA (B(95%CI):−0.12(−0.22 | −0.01), −0.81(−1.07 | −0.55), −1.96(−2.83 | −1.08) respectively; *p* < 0.05 − 0.001) but positively associated with FFMI (B(95%CI) = 0.07(0.02 | 0.13); *p* < 0.01). On the other hand, VPA is positively associated with PBF and VFA (B(95%CI) = 0.72(0.44 | 1), 1.77(0.81 | 2.72) respectively; both *p* < 0.001), negatively with FFMI (B(95%CI)=−0.07(−0.14 | −0.01) * *p* < 0.05). and has no statistically significant association with BMI.

Sex and age of the respondents were also found to be associated with the body composition variables. Girls tend to have significantly higher PBF, but lower FFMI (B(95%CI) = 5.57(4.55 | 6.6), −1.53(−1.74 | −1.31) respectively; all *p* < 0.001) with no statistically significant differences in BMI and VFA. Higher age showed to be associated with higher BMI and FFMI (B(95%CI) = 0.56 (0.41 | 0.7), 0.64(0.57 | 0.71) respectively; both *p* < 0.001) and with lower levels of PBF (B(95%CI) −0.82 (−1.19 | −0.46); *p* < 0.001).

After the involvement of all individual predictors in one regression model (Table 3), only independent and substantial associations remained statistically significant. Eating breakfast during weekdays (B ranged from − 0.06 – −1.4, *p* < 0.05 − 0.01) and sweets consumption (B ranged from − 0.12–0.3.25, *p* < 0.001) remained negatively associated with all body composition variables. Energy drinks consumption remained associated with PBF (B(95%CI)=−0.51(−0.95 | −0.07), *p* < 0.05). MVPA remained negatively associated with PBF and VFA (B(95%CI)=−0.7(−1.01 | −0.39), −1.42(−2.53 | −0.32) respectively; *p* < 0.05 − 0.001) and positively associated with FFMI (B(95%CI) = 0.1 (0.04 | 0.16); *p* < 0.001). The association of MVPA with BMI lost its statistical significance in this regression model. VPA remained positively associated only with VFA (B(95%CI) = 1.45(0.25 | 2.66); *p* < 0.05). Sex and age associations remained similar to the crude effects in previous unadjusted regression model.

**Table 3:** Associations of dietary behaviours, physical activity variables on body composition variables in mutually adjusted regression model (linear regression)

Adjusted model	BMI	PBF	VFA	FFMI
	B (95% C.I.)	B (95% C.I.)	B (95% C.I.)	B (95% C.I.)
**Breakfast weekdays**	−0.16 (−0.28 | −0.05) **	−0.34 (−0.61 | −0.07) *	−1.4 (−2.37 | −0.43) **	−0.06 (−0.11 | 0.00) *
**Family meal**	−0.01 (−0.22 | 0.20)	−0.12 (−0.61 | 0.37)	−0.94 (−2.7 | 0.81)	0.03 (−0.06 | 0.12)
**Fruits**	−0.05 (−0.23 | 0.14)	−0.24 (−0.68 | 0.20)	−1.38 (−2.95 | 0.18)	0.02 (−0.06 | 0.10)
**Vegetables**	0.15 (−0.03 | 0.33)	0.36 (−0.06 | 0.78)	1.66 (0.16 | 3.15) *	0.04 (−0.04 | 0.12)
**Sweets**	−0.42 (−0.57 | −0.27) ***	−0.98 (−1.34 | −0.62) ***	−3.25 (−4.53 | −1.97) ***	−0.12 (−0.19 | −0.05) ***
**Soft drinks**	−0.01 (−0.16 | 0.14)	0.03 (−0.32 | 0.38)	0.25 (−1.00 | 1.5)	−0.02 (−0.09 | 0.05)
**Energy drinks**	−0.05 (−0.24 | 0.14)	−0.51 (−0.95 | −0.07) *	−1.18 (−2.76 | 0.39)	0.07 (−0.01 | 0.16)
**MVPA**	−0.06 (−0.19 | 0.07)	−0.7 (−1.01 | −0.39) ***	−1.42 (−2.53 | −0.32) *	0.1 (0.04 | 0.16) ***
**VPA**	0.07 (−0.07 | 0.22)	0.27 (−0.07 | 0.61)	1.45 (0.25 | 2.66) *	0 (−0.06 | 0.07)
**Sex** (boys vs. girls)	−0.46 (−0.94 | 0.02)	5.11 (3.98 | 6.25) ***	0.32 (−3.72 | 4.36)	−1.36 (−1.58 | −1.15) ***
**Age**	0.58 (0.42 | 0.75) ***	−0.64 (−1.03 | −0.25) ***	0.52 (−0.86 | 1.90)	0.62 (0.55 | 0.70) ***

*BMI* Body mass index, *PBF* Percentage of body fat, *VFA* Visceral fat area, *FFMI* Fat-free mass index (kg/m²), *MVPA* Moderate-to-vigorous physical activity, *VPA* Vigorous physical activity, *B* (95% C.I.) beta coefficients (95% confidence intervals), *p < 0.05, **p < 0.01, ***, *p* < 0.001

## Discussion

A comparison of individual variables between boys and girls in adolescence (Table 1) revealed differences in several areas, namely body composition, dietary behaviours, and physical activity. Differences in body composition parameters between boys and girls – specifically in BMI, PBF, and FFMI – are characteristic of the developmental period of adolescence [28–30]. The World Health Organization and other researchers [31] have highlighted the limitations of assessing overweight and obesity in children based solely on BMI. Classification of normal weight or overweight using BMI may overestimate body fat in children; therefore, it is advisable to improve the accuracy of identifying overweight and obese individuals by using additional indicators such as FFMI or percentage of body fat [32]. The mean BMI value in our sample (20.2) is comparable to findings from other studies [33, 34]. A more accurate indicator of overweight and obesity may be the percentage of body fat (PBF%). Commonly used PBF% cut-off values for obesity, estimated by DEXA in children and adolescents aged 4–18 years, range from ≥ 25% in males to ≥ 30% in females [35]. Studies employing skinfolds as a measure of body fatness have reported identical threshold values [36]. Based on these recommendations, we conclude that the mean PBF% values for both boys and girls in our sample fall within the healthy range.

With regard to dietary behaviours, we observed more frequent breakfast consumption during weekdays, less frequent family meals, but also higher consumption of sweetened beverages and energy drinks among boys. Hammond and Reid [37] suggest that the higher prevalence of energy drink use among boys may be related to masculine advertising and marketing strategies. Thus, gender represents an important factor influencing energy drink consumption. Other contributing factors include the availability of energy drinks at home, peer influence, permissive parental attitudes toward their consumption, perceptions of energy drinks as safe or beneficial, and higher disposable income [38]. Compared to boys, girls were more likely to consume fruits and vegetables, but also sweets. These findings are consistent with previous studies [39, 40].

Regarding the frequency of physical activity, our findings revealed gender differences, with boys spending more time engaged in both MVPA and VPA. This trend among Slovak adolescents has also been confirmed by Madarasová-Gecková et al. [41] and other researchers [39, 42, 43].

The quality of body composition depends on multiple factors such as age, gender [44, 45], eating habits [46], and physical activity [47]. Using crude effect linear regression, we analyzed the influence of individual variables on selected body composition parameters (Table 2). The regression analysis showed that a higher frequency of breakfast consumption during the week was associated with lower BMI, PBF, and VFA values, but also with lower FFMI. This result suggests that regular breakfast consumption reduces the likelihood of overweight and obesity in adolescents. Having breakfast at home may prevent adolescents from selecting foods that do not meet or exceed nutritional recommendations. An inverse relationship between daily breakfast consumption and BMI in adolescents was also reported by Timlin [48] and others [49, 50]. The association between regular breakfast consumption and lower FFMI values in our study may be explained by the fact that a reduction in BMI is often accompanied by a decrease in FFMI. Marshall et al. [51] reported that FMI and FFMI generally increase with BMI percentiles across age groups. A different pattern would likely be observed in athletic populations, where higher BMI values are typically associated with lower percentages of body fat and greater muscle mass [30, 52].

A surprising finding of this study was that more frequent consumption of sweets was associated with lower values of BMI, PBF, VFA, and also FFMI. Although our results contradict those of Oladoyinbo et al. [53], who reported a significant positive association between sweet consumption and body mass index, they are supported by other studies suggesting an inverse relationship between the frequency of sweet intake and BMI or PBF [54–56]. A plausible explanation may lie in the argument that social desirability and perceived appropriateness of dietary behaviours can substantially affect the validity of dietary assessment methods for certain types of foods [57]. Moreover, when using survey- or questionnaire-based dietary assessment methods, adolescents tend to underreport their total energy intake [58]. Therefore, we assume that adolescents with lower weight control or body satisfaction, who are aware that they should limit their sweet consumption, may have reported a lower frequency of sweet intake compared to those who are satisfied with their body weight and have no reason to conceal such behaviour. Similarly, the study by Castillo, Araneda, and Pinheiro [59] found that 69% of children engaged in sports reported consuming sweets daily.

Another interesting finding was that more frequent consumption of energy drinks was associated with lower PBF and higher FFMI values. This result may be explained by the fact that energy drink consumption has become a common dietary habit, particularly among adolescents, young adults, and athletes [60]. Costa, Hayley, and Miller [61] report that the main reasons for energy drink consumption among adolescents include pleasure, functionality (e.g., enhancing sports performance, reducing fatigue, and increasing energy), and social factors. The widespread use of energy drinks among athletes may also be influenced by marketing claims promoting their positive effects on sports performance [37]. Previous research indicates that moderate consumption of energy drinks can improve cognitive abilities and athletic performance [62, 63]. However, excessive caffeine intake is associated with several health risks, including arrhythmias, gastrointestinal disturbances, and dependence [64], as well as increased anxiety and irritability, reduced attention, and reversible cardiovascular events [65]. These potential health risks justify recommendations against the inclusion of energy drinks in the diets of vulnerable populations, particularly adolescents [66], as the adverse effects may not only impair athletic performance but also lead to negative health consequences outside sports contexts [67].

The findings of this study also revealed that MVPA and VPA levels are associated with body composition parameters. Higher levels of MVPA and VPA were associated with lower values of PBF, VFA, and BMI, and with higher FFMI levels. These results are consistent with the findings of Moreno-Díaz, Vaquero-Solís, Tapia-Serrano, and Sánchez-Miguel [43], who confirmed that higher levels of physical activity are linked to lower BMI values. However, mutual adjustment indicated that sweet consumption, MVPA, and breakfast frequency during the week have the most significant effects on BMI, PBF, VFA, and FFMI levels.

The only variable in our study that does not fit the expected concept of a healthy lifestyle and its favourable impact on body composition is the consumption of sweets. As mentioned above, this paradox may be explained by the fact that the majority of physically active adolescents consume sweets daily. Another possible explanation, according to Martinez-Avila et al. [14], is that physically active young adults are more likely to consciously self-report tendencies toward binge eating and uncontrolled eating, possibly perceiving physical activity as a justification or “reward” for such behaviour.

### Limitations

Several limitations of this study should be acknowledged. First, the cross-sectional design does not allow for the establishment of causal relationships. Second, self-reported data on dietary behaviours and physical activity may have introduced bias due to inaccuracies in recall. Third, the analysis is based solely on data from a single Slovak region (the Prešov self-governing region), which limits the generalisability of the findings.

## Conclusion

Despite the global epidemic of overweight and obesity, the analysis of body composition among adolescents in our sample indicated adequate BMI, percentage of body fat and visceral fat area values. Gender-based comparisons of dietary behaviours revealed more frequent consumption of sweetened and energy drinks among boys, whereas girls reported higher intake of fruits, vegetables, and sweets. Boys were also more active in terms of MVPA and VPA. As expected, MVPA and VPA were significantly associated with all body composition parameters. A key finding of the study was that regular breakfast consumption reduced the likelihood of overweight and obesity in adolescents. The findings also draw attention to the frequent consumption of energy drinks, particularly among physically active adolescents. Overall, our results suggest that higher breakfast frequency and engagement in moderate-to-vigorous physical activity are associated with favourable body composition parameters in adolescents.

## Data Availability

The datasets used and/or analysed during the current study are available from the corresponding author on reasonable request.

## Abbreviations

BMI: Body mass index
FFMI: Fat-free mass index (kg/m²)
HBSC: Health Behaviour in School-aged Children
IQR: Interquartile range
M: mean
MVPA: Moderate-to-vigorous physical activity
PA: Physical activity
PBF: Percentage of body fat
SD: Standard deviation
VFA: Visceral fat area
VPA: Vigorous physical activity
